# Enterovirus D68–Associated Respiratory Illness in Children

**DOI:** 10.1001/jamanetworkopen.2025.9131

**Published:** 2025-05-08

**Authors:** Benjamin R. Clopper, Adriana S. Lopez, Leah A. Goldstein, Terry Fei Fan Ng, Ariana P. Toepfer, Mary A. Staat, Elizabeth P. Schlaudecker, Leila C. Sahni, Julie A. Boom, Jennifer E. Schuster, Rangaraj Selvarangan, Natasha B. Halasa, Laura S. Stewart, John V. Williams, Marian G. Michaels, Geoffrey A. Weinberg, Peter G. Szilagyi, Eileen J. Klein, Janet A. Englund, Meredith L. McMorrow, Heidi L. Moline, Claire M. Midgley

**Affiliations:** 1 US Centers for Disease Control and Prevention, Atlanta, Georgia; 2 Cincinnati Children’s Hospital Medical Center, University of Cincinnati College of Medicine, Cincinnati, Ohio; 3 Department of Pediatrics, Baylor College of Medicine, Houston, Texas; 4 Texas Children’s Hospital, Houston; 5 Children’s Mercy Hospital, Kansas City, Missouri; 6 Vanderbilt University Medical Center, Nashville, Tennessee; 7 UPMC Children’s Hospital of Pittsburgh, University of Pittsburgh School of Medicine, Pittsburgh, Pennsylvania; 8 University of Wisconsin School of Medicine and Public Health, Madison; 9 University of Rochester School of Medicine & Dentistry, Rochester, New York; 10 UCLA Mattel Children’s Hospital, Los Angeles, California; 11 Seattle Children’s Research Institute, Seattle, Washington; 12 University of Washington School of Medicine, Seattle; 13 US Public Health Service, Rockville, Maryland

## Abstract

**Importance:**

Enterovirus D68 (EV-D68) typically causes mild to severe acute respiratory illness (ARI). Testing and surveillance for EV-D68 in the US are limited, and important epidemiologic gaps remain.

**Objective:**

To characterize the epidemiology and clinical severity of EV-D68 among US children seeking care for ARI from 2017 to 2022, using a multisite, active, systematic surveillance network.

**Design, Setting, and Participants:**

This cross-sectional study collected data from the New Vaccine Surveillance Network, an active, prospective, population-based surveillance system of emergency departments (EDs) and hospitals at 7 US academic medical centers. Children with ARI and EV-D68–positive results were enrolled during platform-wide EV-D68 testing periods (July to October 2017, July to November 2018, July to November 2020, and July 2021 to December 2022). Included children were aged younger than 18 years, reported 1 or more qualifying ARI symptoms, with a symptom duration less than 14 days at enrollment. Data were analyzed from in October 2024.

**Exposures:**

Laboratory-confirmed EV-D68 infection, including overall infections or those without viral codetection.

**Main Outcomes and Measures:**

Trends and characteristics of EV-D68, including demographics, underlying conditions, and clinical severity by health care setting, were explored. Among hospitalized children with EV-D68–positive results without viral codetection, multivariable logistic regression was used to examine factors associated with receipt of (1) supplemental oxygen or (2) intensive care.

**Results:**

From 2017 to 2022, 976 children with EV-D68–positive results were identified (median [IQR] age, 47 [18-63] months; 391 [40.1%] female); most were enrolled in 2018 (382 children) and 2022 (533 children). Among these, 856 had no viral codetection, of which 320 were discharged home from the ED (median [IQR] age, 33 [16-59] months; 180 male [56.3%]; 237 [74.1%] with no reported underlying conditions) and 536 were hospitalized (median [IQR] age, 40 [19-69] months; 330 male [61.6%]; 268 [50.0%] with no reported underlying conditions). Among those hospitalized, 199 (37.1%) reported a history of asthma or reactive airway disease (RAD) and 77 (14.4%) reported a condition other than asthma or RAD. Having an underlying condition other than asthma or RAD was associated with increased odds of receiving supplemental oxygen (adjusted odds ratio, 2.72; 95% CI, 1.43-5.18) or intensive care admission (adjusted odds ratio, 3.09; 95% CI, 1.72-5.56); neither age group nor history of asthma or RAD were associated with oxygen receipt or intensive care admission.

**Conclusions and Relevance:**

In this cross-sectional study of children with medically attended EV-D68 infections, EV-D68 was associated with severe disease in otherwise healthy children of all ages, and children with nonasthma or RAD comorbidities were at higher risk for severe outcomes when hospitalized.

## Key Points

**Question:**

What are the characteristics and measures of clinical severity of US children with enterovirus D68 (EV-D68)–associated respiratory illness?

**Findings:**

In this cross-sectional study of an active, prospective, respiratory surveillance platform from 2017 to 2022, 976 children with EV-D68–positive results were identified, including 856 children with EV-D68 infections without viral codetection (320 visited an emergency department and 536 were hospitalized); one-half of all hospitalized children had no underlying medical condition, and one-third had a history of asthma. Having a nonasthma underlying medical condition was associated with (1) receiving supplemental oxygen and (2) intensive care admission.

**Meaning:**

These findings suggest that EV-D68 can cause severe disease in otherwise healthy children of all ages and that those with a nonasthma comorbidity may be at higher risk for severe outcomes.

## Teaser

This cross-sectional study analyzes the epidemiology and clinical severity of enterovirus D68–associated respiratory illness among US children seeking care from 2017 to 2022.

## Introduction

Enterovirus D68 (EV-D68) is a nonenveloped RNA virus of the *Enterovirus* genus in the *Picornaviridae* family. First identified in 1962,^1^ EV-D68 infection causes mild to severe acute respiratory illness (ARI), including acute asthma exacerbations.^2,3,4,5,6,7^ Given a common asthma-like presentation, concerns for children with a history of asthma have been appropriately raised; however, there is mixed evidence on prior asthma as a risk factor for more severe EV-D68 illness,^2,3,7^ and little is known about other factors associated with more severe respiratory outcomes. EV-D68 is also associated with acute flaccid myelitis, a rare but serious neurologic condition presenting as limb weakness^4,8,9,10,11,12,13^ with variable recovery of function.

In 2014, the US experienced a nationwide outbreak of severe respiratory illness, with a concomitant increase in acute flaccid myelitis, predominantly among children,^2,8^ highlighting EV-D68 as a cause of pediatric respiratory and neurologic illness. Prior to 2014, EV-D68 surveillance in the US was passive and relied on intermittent EV typing by neutralization or viral sequencing, resulting in sporadic reports of EV-D68 detections or clusters.^14,15,16^ Following the 2014 outbreak, EV-D68 awareness has improved; testing capacity for EV-D68 has expanded to include EV-D68–specific, real-time reverse-transcriptase polymerase-chain-reaction (rRT-PCR) tests^17^; and EV-D68 surveillance was integrated into more health care settings. Since 2014, reports from multiple distinct US sources identified EV-D68 detections in 2014, 2016, 2018, 2020, 2021, and 2022.^18,19,20,21,22,23,24,25,26^ Despite recent advances, EV-D68 testing in the US remains limited to select clinical, public health, or research settings, and EV-D68 reports have been based on single years, local surveillance at single sites with limited numbers to characterize patients, or passively collected or outbreak-driven data. As such, important knowledge gaps remain, and systematic surveillance is needed.

Starting in 2017, EV-D68 surveillance was incorporated into the New Vaccine Surveillance Network (NVSN), an active, prospective platform for ARI across 7 US pediatric medical centers^27^; preliminary EV-D68 data from this platform have been reported previously.^19,23,25^ To provide a systematic overview of EV-D68–associated ARI epidemiology in US children, we used NVSN data to characterize EV-D68 trends and clinical features in children from 2017 to 2022 and assess patient factors associated with more severe EV-D68–associated respiratory illness. These data may inform clinical and public health preparedness.

## Methods

This cross-sectional study was reviewed and approved by the institutional review boards at the Centers for Disease Control and Prevention, Vanderbilt University Medical Center, University of Rochester School of Medicine and Dentistry, Cincinnati Children’s Hospital Medical Center, Seattle Children’s Hospital, Texas Children’s Hospital, Children’s Mercy Hospital, and UPMC Children’s Hospital of Pittsburgh (see 45 CFR § 46 and 21 CFR § 56). All participants and/or their parents or guardians provided written informed consent (or verbal assent, if applicable) prior to enrollment. This work adheres to the Strengthening the Reporting of Observational Studies in Epidemiology (STROBE) reporting guideline for reporting cross-sectional data.^28^

### Platform

NVSN is an active, prospective, population-based surveillance system for ARI at 7 US pediatric medical centers across the US: Cincinnati, Ohio; Houston, Texas; Kansas City, Missouri; Nashville, Tennessee; Pittsburgh, Pennsylvania; Rochester, New York; and Seattle, Washington. Enrollment centers and their catchment areas have been described previously.^29^ Children aged younger than 18 years who presented to an NVSN enrollment facility (emergency department [ED] or inpatient), resided within the defined surveillance area, were experiencing 1 or more qualifying ARI symptoms (apnea, cough, earache, fever, myalgia, nasal congestion, runny nose, sore throat, vomiting after coughing, shortness of breath, wheezing, or apparent life-threatening event or brief resolved unexplained event), and had an illness duration less than 14 days (updated to <10 days beginning November 13, 2022) were eligible for enrollment. Approximately 60% of eligible children approached for enrollment were enrolled between 2016 and 2021^29^; additional details regarding NSVN inclusion and exclusion criteria, methodology, and testing practices are published elsewhere.^29^ For this analysis, we included patients with EV-D68–positive test results who were hospitalized or visited the ED and were discharged home without being hospitalized.

### Data Collection

Patient demographics (including age, sex, and self-reported race and ethnicity) and symptom information were collected during the parent interview. Race and ethnicity were categorized as Black, non-Hispanic; Hispanic; White, non-Hispanic; other, non-Hispanic (ie, any race or ethnicity not otherwise specified), and unknown race or ethnicity. Race and ethnicity were included to included to describe participant demographics and to assess and account for potential differences in health outcomes. Underlying conditions, discharge diagnoses, and measures of clinical severity (oxygen support, length of stay, and receipt of intensive care) were collected through medical chart abstraction.

Trained research staff collected respiratory specimens (midturbinate nasal swabs and/or throat swabs) from enrolled children for pathogen testing by rRT-PCR at each site. EV-D68 testing by rRT-PCR^17^ was introduced to the current 7 NVSN sites in 2017 and was performed seasonally during the summer and fall of 2017 to 2020 to reflect the typical EV season in the US (July to October 2017, July to November 2018, and July to November 2020). Beginning in July 2021, EV-D68 testing was performed year-round to assess detections outside of the EV season and to fully assess EV-D68 trends and seasonality. Sites differed in their EV-D68 testing algorithms (eTable 1 in Supplement 1); in brief, during EV-D68 surveillance periods, 3 NVSN sites tested all ARI specimens for EV-D68, whereas the remaining 4 sites tested specimens already known to be positive for rhinovirus and/or EV.

A convenience sample of available EV-D68–positive respiratory specimens collected in 2018, 2020, 2021, and 2022 was sequenced and analyzed to identify EV-D68 lineage.^30^ Clade determination was performed by next generation sequencing and comparing the VP1 region.

### Statistical Analysis

We described EV-D68 trends, patient demographics, and measures of clinical severity of patients with EV-D68–positive results by health care setting and viral codetection status. When assessing EV-D68 detections over time, we reported detections by surveillance year or dichotomized as pre–COVID-19 pandemic (start of surveillance on July 1, 2017, to February 29, 2020) or COVID-19 pandemic (March 1, 2020, to December 31, 2022). To explore factors associated with severe EV-D68–associated respiratory illness, we compared differences in EV-D68 characteristics among hospitalized children using 2 analytic outcomes: (1) receipt of supplemental oxygen or (2) treatment in the intensive care unit (ICU). Patients with viral codetections were excluded to better understand EV-D68–specific clinical presentations. We used Pearson χ^2^ tests of association for comparison of categorical variables and the Wilcoxon rank-sum test for comparison of age because these data were continuous and not normally distributed. We used .05 as a threshold of statistical significance.

We next used multivariable logistic regression to estimate the odds of requiring supplemental oxygen vs not (referent), and separately, the odds of receiving ICU care vs not (referent), using the following demographic and clinical exposure variables: dichotomized COVID-19 pandemic period, age group, sex, race and ethnicity, study site, history of asthma or reactive airway disease (RAD), and history of any underlying condition other than asthma or RAD (ie, a nonasthma underlying condition). Patients with viral codetections were excluded for the main analysis but were included in a sensitivity analysis. Because diagnosing asthma and RAD is challenging in children younger than 2 years, we performed a sensitivity analysis restricted to children ages 2 to 17 years. Statistical analyses were performed using SAS software version 9.4 (SAS Institute) in October 2024.

## Results

### EV-D68 Detections and Trends Across Multiple Years

**Figure 1.**
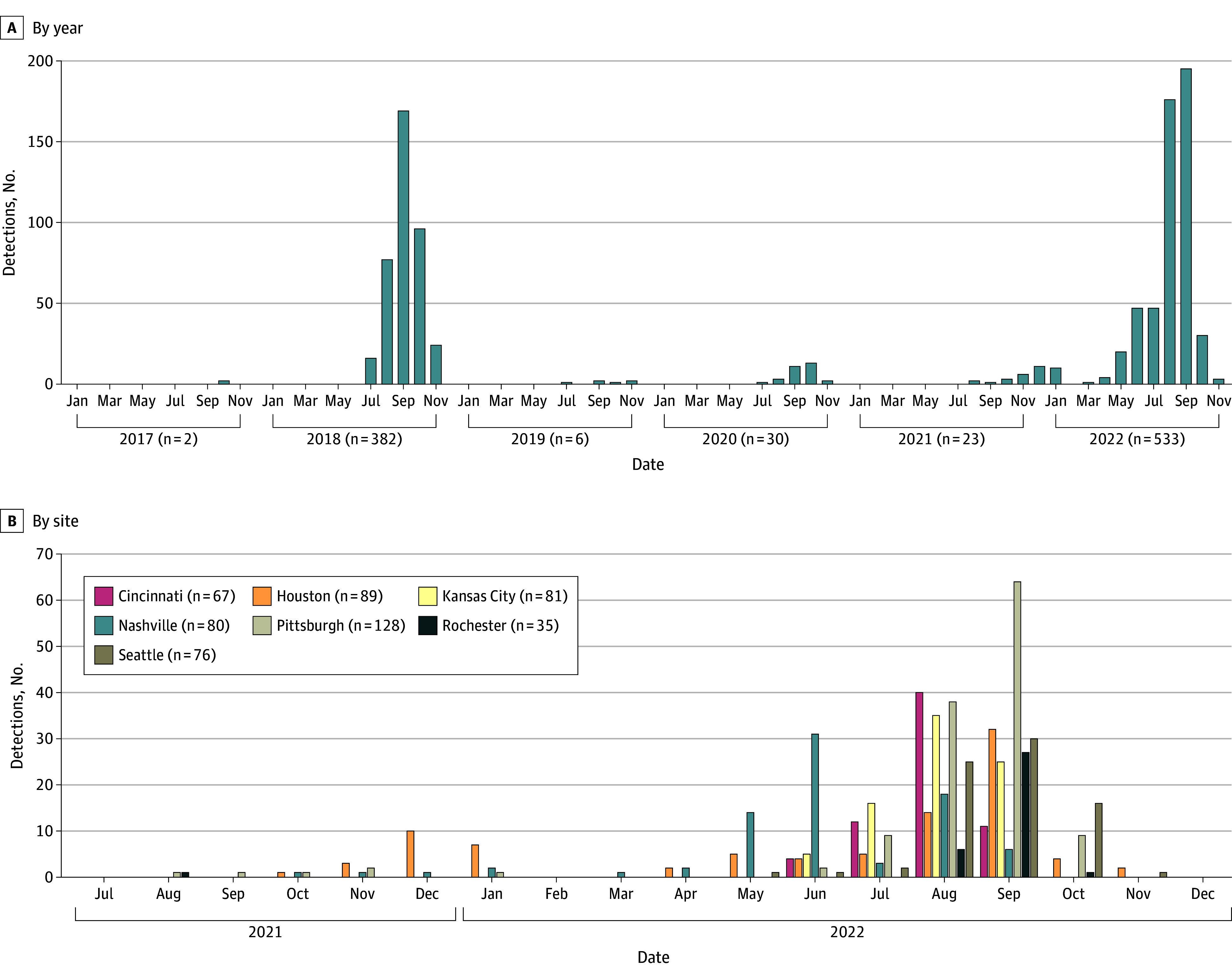
Enterovirus-D68 Detections by Year and Site, New Vaccine Surveillance Network, 2017-2022

**Figure 2.**
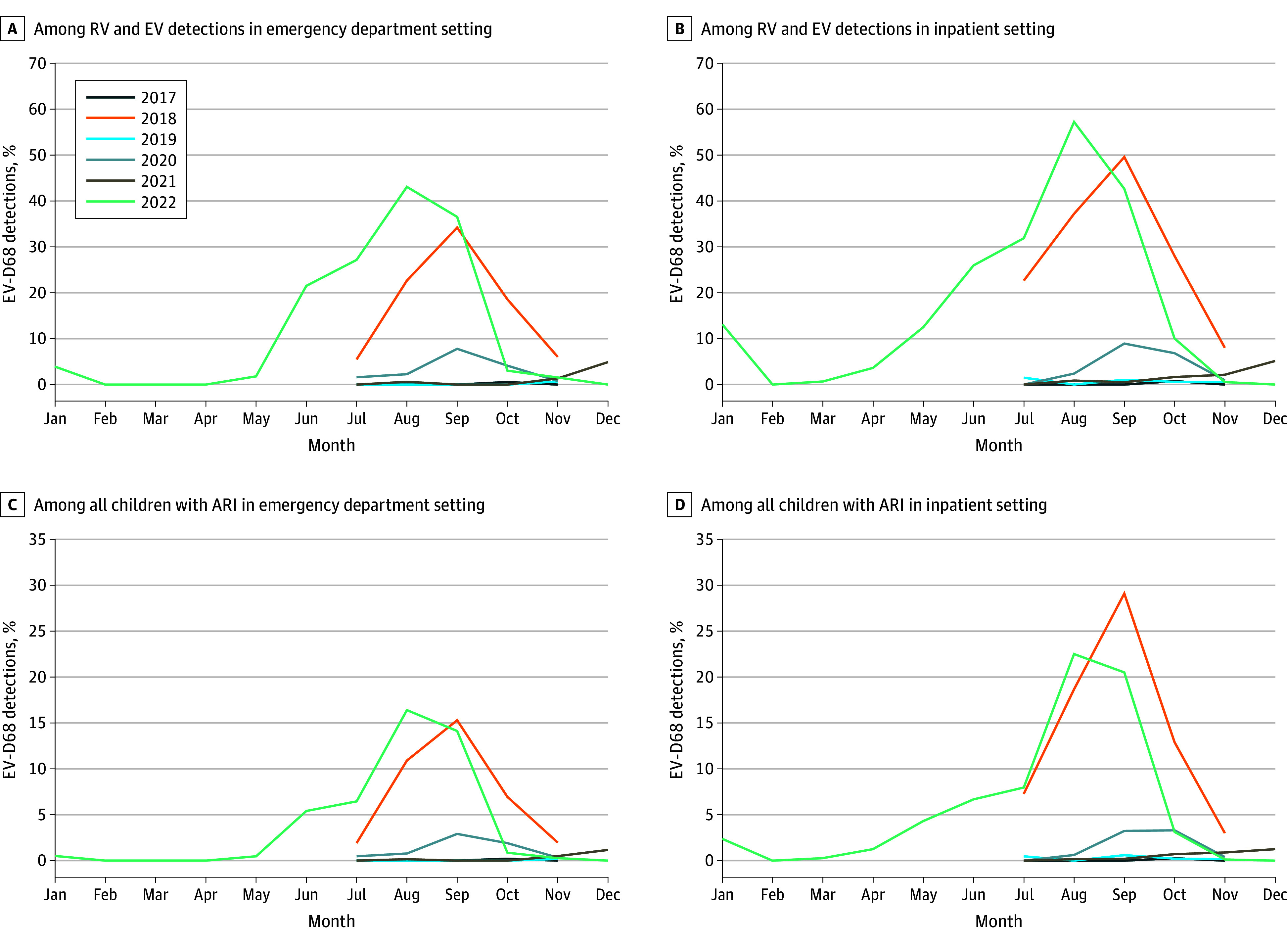
Enterovirus D68 (EV-D68) Detections Among Rhinovirus (RV) and EV Positive Cases and All Acute Respiratory Infection (ARI) Cases by Care Setting, New Vaccine Surveillance Network (2017-2022)

From July 2017 to December 2022, we identified 976 children with EV-D68–positive results (median [IQR] age, 47 [18-63] months; 391 female [40.1%]) from 30 435 children with ARI enrolled during EV-D68 surveillance periods (eTable 2 in Supplement 1). Annual detections during EV-D68 surveillance periods were as follows: 2 detections in 2017, 382 detections in 2018, 6 detections in 2019, 30 detections in 2020, 23 detections in 2021, and 533 detections in 2022 (Figure 1A). More than 90% of EV-D68 detections (915 of 976 detections [92%]) occurred in 2018 or 2022. Detections in both years peaked during September (Figure 1A), although percentage positivity in 2022 peaked earlier in August, and the rise in detections may have been slightly earlier in 2022 than in 2018. In Houston, Nashville, and Pittsburgh, we detected a low level of circulation in late fall and winter of 2021 that continued into the spring of 2022 (Figure 1B). Temporal trends in EV-D68 detection were similar in ED and inpatient settings (Figure 2). Viruses sequenced in 2020 (15 sequenced of 25 attempted) were of lineage A2/D, while viruses sequenced in 2018 (42 of 60 viruses, as previously reported^25^), 2021 (5 of 15 viruses) and 2022 (159 of 223 viruses) were of lineage B3 (eTable 3 in Supplement 1). Sequences from some samples were not sufficient for clade determination, especially in samples with low numbers of viral copies.

### EV-D68–Associated ED Visits

Among 364 children with EV-D68–positive results who were discharged from the ED across the full period, 44 (12.1%) had a viral codetection (Table 1). Among the 320 children without a viral codetection, the median (IQR) age was 33 (16-59) months, 180 (56.3%) were male, 72 (22.5%) were Hispanic, 141 (44.1%) were non-Hispanic Black, and 69 (21.6%) were non-Hispanic White. Most (237 children [74.1%]) had no underlying conditions, while 60 (18.8%) had a history of asthma or RAD (55 children aged 2 to 17 years [27.0%]), and 23 (7.2%) reported a nonasthma underlying condition (Table 2). The most common parent-reported symptoms were cough (301 children [94.1%]), nasal congestion (280 children [87.5%]), and rapid or shallow breathing or shortness of breath (217 children [67.8%]); wheezing and measured fever were reported for 140 children (43.8%) and 187 children (58.4%), respectively. The most common primary discharge diagnoses (based on *International Statistical Classification of Diseases, Tenth Revision, Clinical Modification *[*ICD-10-CM*]) in the ED were asthma-related (67 children [21.0%] with asthma or RAD as the primary diagnosis; 81 children [25.4%] with any diagnosis of asthma or RAD), upper respiratory infection (67 children [21.0%]), or respiratory signs and symptoms (ie, abnormalities of breathing such as wheezing, cough, or shortness of breath; 53 children [16.6%]).

**Table 1.** Demographic Characteristics of Children With EV-D68, by Viral Codetection Status and Health Care Setting, Enrolled in the New Vaccine Surveillance Network (2017-2022)^a^

Characteristic	Participants (all years), No. (%)			
	Single EV-D68 detections only (ie, no viral codetection)		Any EV-D68 detection, including those with viral codetections	
	ED (n = 320)	Inpatient (n = 536)	ED (n = 364)	Inpatient (n = 612)
Viral codtections	NA	NA	44 (12.1)	76 (12.4)
Age group				
Median (IQR), mo	33 (16-59)	40 (19-69)	32 (16-58)	37 (18-67)
0-5 mo	24 (7.5)	19 (3.5)	26 (7.1)	27 (4.4)
6-11 mo	37 (11.6)	39 (7.3)	43 (11.8)	45 (7.4)
12-23 mo	55 (17.2)	115 (21.5)	68 (18.7)	144 (23.5)
24-59 mo	125 (39.1)	195 (36.4)	143 (39.3)	216 (35.3)
5-11 y	58 (18.1)	145 (27.1)	63 (17.3)	156 (25.5)
12-17 y	21 (6.6)	23 (4.3)	21 (5.8)	24 (3.9)
Sex				
Female	140 (43.8)	206 (38.4)	151 (41.5)	240 (39.2)
Male	180 (56.3)	330 (61.6)	213 (58.5)	372 (60.8)
Race and ethnicity				
Black, non-Hispanic	141 (44.1)	182 (34.0)	164 (45.1)	203 (33.2)
Hispanic	72 (22.5)	94 (17.5)	74 (20.3)	110 (18.0)
White, non-Hispanic	69 (21.6)	199 (37.1)	86 (23.6)	232 (37.9)
Other, non-Hispanic^b^	34 (10.6)	55 (10.3)	36 (9.9)	60 (9.8)
Unknown	4 (1.3)	6 (1.1)	4 (1.1)	7 (1.1)
Surveillance year				
2017 (July-October)	1 (0.3)	1 (0.2)	1 (0.3)	1 (0.2)
2018 (July-November)	115 (35.9)	233 (43.5)	125 (34.3)	257 (42.0)
2019 (July-November)	1 (0.3)	4 (0.8)	1 (0.3)	5 (0.8)
2020 (July-November)	15 (4.7)	15 (2.8)	15 (4.2)	15 (2.5)
2021 (July-December)	7 (2.2)	13 (2.4)	8 (2.2)	15 (2.5)
2022 (January-December)	181 (56.6)	270 (50.4)	214 (58.8)	319 (52.1)
Surveillance period^c^				
Pre–COVID-19 pandemic	117 (36.6)	238 (44.4)	127 (34.9)	263 (43.0)
During COVID-19 pandemic	203 (63.4)	298 (55.6)	237 (65.1)	349 (57.0)
New Vaccine Surveillance Network site				
Nashville, Tennessee	52 (16.3)	65 (12.1)	59 (16.2)	75 (12.3)
Rochester, New York	30 (9.4)	64 (11.9)	32 (8.8)	68 (11.1)
Cincinnati, Ohio	39 (12.2)	67 (12.5)	46 (12.6)	80 (13.1)
Seattle, Washington	44 (13.8)	55 (10.3)	52 (14.3)	64 (10.5)
Houston, Texas	33 (10.3)	77 (14.4)	35 (9.6)	88 (14.4)
Kansas City, Missouri	74 (23.1)	67 (12.5)	79 (21.7)	73 (11.9)
Pittsburgh, Pennsylvania	48 (15.0)	141 (26.3)	61 (16.8)	164 (26.8)

Abbreviations: ED, emergency department; EV-D68, enterovirus-D68; NA, not applicable.

^a^ Includes data recorded in EV-D68 surveillance months during 2017 to 2022 period: July 1, 2017, to October 31, 2017; July 1, 2018, to November 30, 2020; and July 1, 2021, to December 31, 2022. Percentages may not add to 100 due to rounding.

^b^ Study participants indicated other, non-Hispanic race and ethnicity voluntarily if they did not self-identify as Black, non-Hispanic; Hispanic; or White, non-Hispanic race and ethnicity.

^c^ The pre–COVID-19 pandemic period was defined as January 1, 2017, to February 29, 2020. The COVID-19 pandemic period was defined as March 1, 2020, to December 31, 2022.

**Table 2.** Clinical Characteristics of Children With EV-D68, by Viral Codetection Status and Health Care Setting, Enrolled in the New Vaccine Surveillance Network (2017-2022)^a^

Characteristics	Participants (all years), No. (%)			
	Single EV-D68 detections only (ie, no viral codetection)		Any EV-D68 detection, including those with viral codetections	
	ED (n = 320)	Inpatient (n = 536)	ED (n = 364)	Inpatient (n = 612)
Viral codetections	NA	NA	44 (12.1)	76 (12.4)
Underlying medical conditions^b^				
No underlying condition	237 (74.1)	268 (50.0)	273 (75.0)	311 (50.8)
≥1 Underlying condition	83 (25.9)	268 (50.0)	91 (25.0)	301 (49.2)
Any underlying condition (excluding asthma and RAD)	23 (7.2)	77 (14.4)	25 (6.9)	88 (14.4)
Asthma or RAD	60 (18.8)	199 (37.1)	65 (17.9)	223 (36.4)
Asthma and RAD (among ages 2-17 y only)	55 (27.0)	182 (50.1)	58 (25.6)	199 (50.3)
Chronic lung disease (excluding asthma and RAD)^c^	4 (1.3)	18 (3.4)	4 (1.1)	20 (3.3)
Neuromuscular disease^d^	8 (2.5)	31 (5.8)	9 (2.5)	33 (5.4)
Immunocompromised^e^	2 (0.6)	6 (1.1)	2 (0.6)	7 (1.1)
Heart condition^f^	7 (2.2)	19 (3.5)	7 (1.9)	22 (3.6)
Developmental disorder^g^	3 (2.6)	30 (9.2)	4 (1.1)	31 (5.1)
Severity of illness				
Length of stay, d				
Mean	NA	2.0 (2.7)	NA	2.0 (2.7)
Median (IQR)	NA	1.0 (1-2)	NA	1.0 (1-2)
Respiratory support				
Supplemental oxygen	32 (10.0)	339 (63.1)	35 (9.7)	385 (62.9)
Intubation	0	11 (2.1)	0	13 (2.1)
Intensive care unit	0	87 (16.2)	0	105 (17.2)
Death	0	0	0	0
Symptom presentation^h^				
Cough	301 (94.1)	518 (96.6)	342 (94.0)	593 (96.9)
Nasal congestion	280 (87.5)	441 (82.3)	322 (88.5)	512 (83.7)
Wheezing	140 (43.8)	418 (78.0)	157 (43.1)	472 (77.1)
Fever	187 (58.4)	295 (55.0)	220 (60.4)	341 (55.7)
Ear pain	57 (17.8)	50 (9.3)	63 (17.4)	63 (10.3)
Sore throat	84 (26.3)	140 (26.1)	92 (25.3)	154 (25.2)
Vomiting	43 (13.4)	89 (16.6)	49 (13.5)	103 (16.8)
Muscle pain	33 (10.3)	54 (10.1)	38 (10.5)	63 (10.3)
Rapid or shallow breathing or shortness of breath	217 (67.8)	503 (93.8)	248 (68.1)	575 (94.0)
Asthma-related discharge diagnosis codes^i^				
Primary asthma or RAD	67 (21.0)	266 (49.6)	73 (20.2)	290 (47.4)
Any asthma or RAD	81 (25.4)	333 (62.1)	88 (24.3)	367 (60.0)
Alternate specific	67 (21.0)	292 (54.5)	71 (19.6)	320 (52.3)
Other primary discharge diagnosis codes				
Upper respiratory infection^j^	67 (21.0)	24 (4.5)	79 (21.8)	25 (4.1)
Bronchiolitis (*ICD-10-CM* code J.21)	18 (5.6)	73 (13.6)	24 (6.6)	94 (15.4)
Respiratory signs and symptoms^k^	53 (16.6)	38 (7.1)	56 (15.5)	42 (6.9)
Wheezing	10 (3.1)	14 (2.6)	11 (3.0)	15 (2.5)
Fever	19 (6.0)	2 (0.4)	21 (5.8)	3 (0.5)
Pneumonia (nontuberculosis)^l^	6 (1.9)	37 (6.9)	6 (1.7)	40 (6.5)

Abbreviations: ED, emergency department; EV-D68, enterovirus-D68; *ICD-10-CM*, *International Statistical Classification of Diseases, Tenth Revision, Clinical Modification*; NA, not applicable; RAD, reactive airway disease.

^a^ Includes data recorded in EV-D68 surveillance months during 2017 to 2022 period: July 1, 2017, to October 31, 2017; July 1, 2018, to November 30, 2020; and July 1, 2021, to December 31, 2022.

^b^ Included congenital heart malformation or other heart condition, transplant recipient, cancer, sickle cell anemia, cerebral palsy, seizure disorder or other neurologic or neuromuscular disorder, asthma or RAD, cystic fibrosis, bronchopulmonary dysplasia, chronic lung disease of prematurity or other chronic lung condition, kidney disease, Down syndrome or other genetic or metabolic disorder, blood disorders, liver disease, diabetes, chronic endocrine condition, chronic gastrointestinal disease, and other developmental disabilities.

^c^ Cystic fibrosis, bronchopulmonary dysplasia, chronic lung disease of prematurity, or other chronic lung condition.

^d^ Cerebral palsy, seizure disorder, or other neurologic or neuromuscular disorder.

^e^ Immune condition, transplant recipient (peripheral blood stem cells, bone marrow, cord blood, or organ), cancer, and sickle cell anemia.

^f^ Congenital heart malformation or other heart condition.

^g^ Including intellectual disability, pervasive developmental disorder, global developmental delay, Autism spectrum disorder, or other developmental disorder.

^h^ These data were collected during the parent or guardian interview. The researchers’ ability to capture certain symptoms may be dependent on the age of the child. The presence of 1 or more symptoms was required for enrollment.

^i^ Primary diagnosis included *ICD-10-CM* code J45 in the primary position. Any diagnosis included *ICD-10-CM* code J45 in any primary or secondary discharge diagnosis code position (up to 10 codes reported). Alternate specific included cases with discharge diagnosis code J45 and the following subspecifications in any diagnosis code position (primary or secondary): .01, .02, .11, .12, .21, .22, .31, .32, .41, .42, .51, .52, .901, and .902.

^j^ *ICD-10-CM *codes included J02.0, J02.8, J02.9, J04.10, J05.0, and J06.9.

^k^ *ICD-10-CM *codes included R05, R05.8, R05.9, R06.00, R06.02, R06.2, R06.03, R06.89, R06.9, R09.02, and R09.81.

^l^ *ICD-10-CM *codes included J12.3, J12.89, J12.9, J15.7, J15.9, J16.8, J18.1, J18.8, and J18.9.

### EV-D68–Associated Hospitalizations

Among 612 inpatients with EV-D68–positive results, only 76 (12.4%) had a viral codetection (Table 1). Among the 536 hospitalized children without a viral codetection, the median (IQR) age was 40 (19-69) months, 330 (61.6%) were male, 94 (17.5%) were Hispanic, 182 (34.0%) were non-Hispanic Black, and 199 (37.1%) were non-Hispanic White. One-half of the hospitalized children (268 children [50.8%]) were previously healthy; one-third (199 children [37.1%]) had a history of asthma or RAD, and 77 (14.4%) had another (nonasthma) underlying condition (Table 2). The most common parent-reported symptoms were cough (518 children [96.6%]), rapid or shallow breathing or shortness of breath (503 children [93.8%]), and nasal congestion (441 children [82.3%]). Wheezing was reported for 418 children (78.0%) and fever was reported for 295 children (55.0%). Almost two-thirds of the hospitalized children (339 children [63.1%]) received oxygen support, 87 (16.2%) received ICU care, and 11 (2.1%) were mechanically ventilated; no children died. The most common primary discharge diagnoses were asthma-related (266 children [49.6%] with asthma or RAD as the primary diagnosis; 333 children [62.1%] with any diagnosis of asthma or RAD), bronchiolitis (73 children [13.6%]), or respiratory signs and symptoms (38 children [7.1%]).

As noted, among the 536 inpatients with EV-D68–positive results without viral codetection, many did not have a prior history of asthma or RAD (337 children [62.9%]), although a history of asthma or RAD was more commonly reported for children aged 5 to 17 years (101 of 168 children [60.1%]) than children aged 2 to 4 years (81 of 195 children [41.5%]) or younger than 2 years (17 of 173 children [9.8%]) (eTable 4 in Supplement 1). In all age groups, the proportion of hospitalized children that required supplemental oxygen therapy was similar between those with (121 of 199 children [60.8%]) and without a reported history of asthma or RAD (218 of 337 children [64.7%]); median (IQR) length of stay was 1 day (1-2 days) for all age groups. Among children aged 2 to 5 years and 5 to 17 years without a prior history of asthma or RAD, most (X of Y children [62.3%] and X of Y children [70.2%], respectively) received an asthma-related discharge diagnosis for their EV-D68–associated hospitalization (eTable 4 in Supplement 1). In children younger than 2 years without a prior history of asthma or RAD, the most common primary discharge diagnosis was bronchiolitis (X of Y children [42.3%]).

### Factors Associated With Severity Among Hospitalized Children

We assessed demographic and clinical factors using 2 markers of severe disease: (1) receipt of supplemental oxygen and (2) ICU care. Among hospitalized children, 339 (63.3%) received supplemental oxygen, and 87 (16.2%) received ICU care. Results from unadjusted analyses are presented in eTable 5 in Supplement 1. In multivariable analyses (Table 3), the presence of a nonasthma underlying condition was associated with an approximate 3-fold increase in the odds of receiving supplemental oxygen (adjusted odds ratio [aOR], 2.72; 95% CI, 1.43-5.18) or receiving ICU care (aOR, 3.09; 95% CI, 1.72-5.56). We did not observe an association of a history of asthma or RAD with oxygen requirement (aOR, 1.17; 95% CI, 0.75-1.84) or ICU care (aOR, 0.87; 95% CI, 0.49-1.56), and we did not observe associations with age group. Children hospitalized during the COVID-19 pandemic period had higher odds of receiving oxygen as compared with the pre–COVID-19 pandemic period (aOR, 1.61; 95% CI, 1.09-2.38); no difference in ICU utilization was observed in relation to COVID-19 pandemic period (Table 3). We observed some differences by site in oxygen receipt. Odds of intensive care receipt were higher for females than males (aOR, 1.75; 95% CI, 1.08-2.86).

**Table 3.** Multivariable Comparison of Supplemental Oxygen Requirement and ICU Receipt Among Hospitalized Children With Enterovirus-D68, New Vaccine Surveillance Network (2017-2022)^a^

Characteristic	Supplemental oxygen				ICU			
	No, No./Total No. (%)	Yes, No./Total No. (%)	OR (95% CI)	aOR (95% CI)	No, No./Total No. (%)	Yes, No./Total No. (%)	OR (95% CI)	aOR (95% CI)
Total	197/536 (36.8)	339/536 (63.2)	NA	NA	449/536 (83.8)	87/536 (16.2)	NA	NA
Surveillance period^b^								
Pre–COVID-19 pandemic	104/238 (43.7)	134/238 (56.3)	1 [Reference]	1 [Reference]	202/238 (84.9)	36/238 (15.1)	1 [Reference]	1 [Reference]
COVID-19 Pandemic	93/298 (31.2)	205/298 (68.8)	1.71 (1.20-2.44)^c^	1.61 (1.09-2.38)^c^	247/298 (82.9)	51/298 (17.1)	1.16 (0.73-1.85)	1.09 (0.65-1.81)
Age group, mo								
0 to <12	23/58 (39.7)	35/58 (60.3)	0.87 (0.47-1.60)	0.80 (0.39-1.65)	49/58 (84.5)	9/58 (15.5)	0.92 (0.41-2.08)	0.75 (0.30-1.87)
12 to <24	37/115 (32.2)	78/115 (67.8)	1.20 (0.73-1.99)	1.39 (0.77-2.50)	92/115 (80.0)	23/115 (20.0)	1.25 (0.68-2.30)	1.34 (0.67-2.65
24 to <60	76/195 (39.0)	119/195 (61.0)	0.89 (0.58-1.37)	0.94 (0.59-1.50)	168/195 (86.2)	27/195 (13.8)	0.80 (0.45-1.43)	0.76 (0.42-1.41)
≥60	61/168 (36.3)	107/168 (63.7)	1 [Reference]	1 [Reference]	140/168 (83.3)	28/168 (16.7)	1 [Reference]	1 [Reference]
Sex								
Female	72/206 (35.0)	134/206 (65.0)	1.14 (0.79-1.63)	1.07 (0.72-1.59)	163/206 (79.1)	43/206 (20.9)	1.72 (1.08-2.72)^c^	1.75 (1.08-2.86)^c^
Male	125/330 (37.9)	205/330 (62.1)	1 [Reference]	1 [Reference]	286/330 (86.7)	44/330 (13.3)	1 [Reference]	1 [Reference]
Race and ethnicity								
Black, non-Hispanic	82/182 (45.1)	100/182 (54.9)	0.66 (0.44-1.00)	0.63 (0.39-1.00)	153/182 (84.1)	29/182 (15.9)	1.03 (0.59-1.78)	1.26 (0.68-2.33)
Hispanic	27/94 (28.7)	67/94 (71.3)	1.35 (0.79-2.30)	0.74 (0.39-1.42)	78/94 (83.0)	16/94 (17.0)	1.11 (0.57-2.15)	0.74 (0.33-1.66)
White, non-Hispanic	70/199 (35.2)	129/199 (64.8)	1 [Reference]	1 [Reference]	168/199 (84.4)	31/199 (15.6)	1 [Reference]	1 [Reference]
Other, non-Hispanic^d^	17/55 (30.9)	38/55 (69.1)	1.21 (0.64-2.30)	1.44 (0.73-2.83)	45/55 (81.8)	10/55 (18.2)	1.20 (0.55-2.64)	1.25 (0.55-2.86)
Unknown	1/6 (16.7)	5/6 (83.3)	2.71 (0.31-23.7	2.81 (0.31-25.4)	5/6 (83.3)	1 (16.7)	1.08 (0.12-9.60)	0.94 (0.09-9.62)
New Vaccine Surveillance Network site								
Nashville, Tennessee	29/65 (44.6)	36/65 (55.4)	0.68 (0.38-1.24)	0.66 (0.35-1.25)	53/65 (81.5)	12/65 (18.5)	1.29 (0.59-2.82)	1.30 (0.58-2.93)
Rochester, New York	30/64 (46.9)	34/64 (53.1)	0.62 (0.34-1.14)	0.82 (0.43-1.56)	57/64 (89.1)	7/64 (10.9)	0.70 (0.28-1.75)	0.86 (0.33-2.22)
Cincinnati, Ohio	39/67 (58.2)	28/67 (41.8)	0.39 (0.22-0.72)^c^	0.49 (0.26-0.91)^c^	58/67 (86.6)	9/67 (13.4)	0.89 (0.38-2.06)	0.87 (0.36-2.11)
Seattle, Washington	24/55 (43.6)	31/55 (56.4)	0.71 (0.38-1.34)	0.58 (0.29-1.13)	46/55 (83.6)	9/55 (16.4)	1.12 (0.48-2.62)	1.20 (0.49-2.96)
Houston, Texas	10/77 (13.0)	67/77 (87.0)	3.68 (1.74-7.78)^c^	3.62 (1.56-8.44)^c^	56/77 (72.7)	21/77 (27.3)	2.14 (1.08-4.24)^c^	2.00 (0.87-4.61)
Kansas City, Missouri	15/67 (22.4)	52/67 (77.6)	1.91 (0.98-3.72)	2.44 (1.20-4.94)	59/67 (76.6)	8/67 (11.9)	0.78 (0.32-1.85)	0.77 (0.31-1.93)
Pittsburgh, Pennsylvania	50/141 (35.5)	91/141 (64.5)	1 [Reference]	1 [Reference]	120/141 (85.1)	21/141 (14.9)	1 [Reference]	1 [Reference]
Any underlying medical conditions (excluding asthma or RAD history)								
No	183/459 (39.9)	276/459 (60.1)	1 [Reference]	1 [Reference]	398/459 (86.7)	61/459 (13.3)	1 [Reference]	1 [Reference]
Yes	14/77 (18.2)	63/77 (81.8)	2.98 (1.62-5.48)^c^	2.72 (1.43-5.18)^c^	51/77 (66.2)	26/77 (33.8)	3.33 (1.93-5.73)^c^	3.09 (1.72-5.56)^c^
History of asthma or RAD								
No	119/337 (35.3)	218/337 (64.7)	1 [Reference]	1 [Reference]	277/337 (82.2)	60/337 (17.8)	1 [Reference]	1 [Reference]
Yes	78/199 (39.2)	121/199 (60.8)	0.85 (0.59-1.22)	1.17 (0.75-1.84)	172/199 (86.4)	27/199 (13.6)	0.73 (0.44-1.19)	0.87 (0.49-1.56)

Abbreviations: aOR, adjusted odds ratio; ICU, intensive care unit; OR, odds ratio; RAD, reactive airway disease.

^a^ Each outcome was adjusted for surveillance period, age group, sex, race and ethnicity, New Vaccine Surveillance Network site, presence of any underlying medical condition (excluding asthma or RAD), and history of asthma or RAD. Cases with viral codetections were excluded. Percentages may not add to 100 due to rounding.

^b^ Pre–COVID-19 pandemic includes surveillance periods from July 2017 to February 2020. The COVID-19 pandemic includes surveillance periods from March 2020 to December 2022.

^c^ Statistically significant.

^d^ Study participants indicated other, non-Hispanic race and ethnicity voluntarily if they did not self-identify as Black, non-Hispanic; Hispanic; or White, non-Hispanic race and ethnicity.

We observed similar associations with severity in sensitivity analyses restricted to children aged 2 to 17 years (eTable 6 in Supplement 1) or including patients (all ages) with viral codetections (eTable 7 in Supplement 1); in the latter, we did not observe a statistically significant association of viral codetection with receipt of supplemental oxygen (aOR, 0.87; 95% CI, 0.51-1.49) or ICU (aOR, 1.52; 95% CI, 0.82-2.80), although codetections were low in number and we may not have been powered to detect a difference.

## Discussion

In this multisite, cross-sectional study of more than 950 US children with medically attended EV-D68–associated respiratory illness from 2017 to 2022, we found that almost one-half of the children hospitalized with EV-D68 had no prior underlying conditions, indicating EV-D68 as a cause of severe respiratory illness in otherwise healthy children in our sample. Most children with EV-D68–positive results received an asthma-related diagnosis, including those with or without a prior history of asthma or RAD. Once hospitalized, children with an underlying condition other than asthma or RAD were at higher risk for receipt of supplemental oxygen or intensive care, potentially informing prevention or clinical triaging strategies for these children.

Following the previously reported low circulation of EV-D68 in 2020 in the NVSN,^25^ we now report unexpected although low EV-D68 circulation during late 2021, consistent with other findings of limited EV-D68 circulation during the same period.^26^ Detections in 2022 were high, possibly beginning slightly earlier and peaking at slightly higher levels than observed in prior years, although year-round surveillance was not available in earlier years. Viral lineages in 2021 and 2022 were the same as those detected in 2018. The seemingly atypical EV-D68 circulation in 2021 and high emergence in 2022 may reflect a post–COVID-19 pandemic viral resurgence, as has been observed for other respiratory viruses,^31,32^ and is consistent with EV-D68 modeling predictions.^33^ These trends, combined with a higher oxygen requirement among children hospitalized with EV-D68 during the COVID-19 pandemic, may reflect an immunity gap following limited circulation since 2018.^34^ However, we did not observe an association of COVID-19 pandemic period with ICU admission, and more work is needed to compare EV-D68 detections between these 2 time periods. It is also possible that the increase in oxygen use observed during the COVID-19 pandemic may reflect changes in the standard of clinical care for respiratory illness, rather than an increase in severity.

A history of asthma or RAD was reported by approximately one-fifth of children with an EV-D68–associated ED visit, and one-third of hospitalized children, which was lower than anticipated based on a previous US report from 2014^2^; this difference may be due to multiple factors, including differences in data collection (active vs passive) or differences in the populations affected over time. Yet, the proportion of children with EV-D68–positive results with a history of asthma or RAD in our current analysis was still notable and higher than the reported prevalence of asthma in the general US population, which is approximately 9.6% for persons younger than 18 years.^35^ Although not the focus of this analysis, this finding suggests that a history of asthma may be associated with a higher risk for EV-D68–associated medically attended illness. Indeed, previous reports suggest that children with EV-D68–associated ARI are more likely to have a history of asthma than children with other respiratory viruses.^3,5,36^ However, once hospitalized, we did not observe an association of a history of asthma or RAD with more severe illness, as defined by either oxygen requirement or ICU admission; this is inconsistent with previous summaries of passively collected US data,^2^ but consistent with a report based on active surveillance.^3^ Our study did not include medications administered, including receipt and timing of treatments for severe asthma-related visits, which may have impacted our ability to robustly assess factors associated with severe asthma exacerbations. More data are needed to better understand the association of a history of asthma with severe EV-D68–associated presentations.

Despite a low report of prior asthma in our study, nearly two-thirds of hospitalized children with or without a history of asthma received an asthma-related discharge diagnosis. The observed asthma-like presentation in children without a prior asthma diagnosis is consistent with previous epidemiologic reports,^6^ and translational evidence that EV-D68 may be associated with interleukin-17–dependent airway inflammation and hyperresponsiveness.^37^ Longitudinal follow up of children with EV-D68–associated illness is needed to better understand if children with an asthma-like presentation subsequently receive a formal diagnosis of asthma, or if these presentations are transient. Although less frequent, children with EV-D68–positive results received a variety of other respiratory-related discharge diagnoses, including bronchiolitis (especially in children <2 years), pneumonia, or general respiratory signs and symptoms (eg, wheezing or cough) indicating the potential for varied presentations. Diagnoses varied by health care setting, age, or prior asthma status.

Both oxygen receipt and ICU admission were associated with a history of a nonasthma medical condition, highlighting these children as being of higher risk for severe illness once hospitalized, which is consistent with observations for other respiratory viruses. We did not observe an association of age with more severe illness, which is unlike observations for some other respiratory viruses,^38,39,40^ highlighting a risk for EV-D68–associated severe disease in children of all ages. Overall, the length of hospitalization was typically short compared with other respiratory viruses.^41,42^

### Limitations

This work is subject to limitations. First, patient enrollment was restricted to ED and inpatient settings, overrepresenting children with severe disease; more work is needed to understand the epidemiology of EV-D68 in less acute settings. Second, supplemental oxygen may not be indicated for some respiratory presentations, such as in older children presenting with an asthma exacerbation, meaning that the factors we describe as being associated with increased oxygen use may reflect standard of care by age or presentation, and not necessarily severity risk. Relatedly, data on medications received may have further informed illness presentation. Third, given the small number of children receiving intensive care, we may have been underpowered to detect some smaller-magnitude associations. Fourth, children without a history of asthma or RAD may have been misclassified based on inaccurate reports from caregivers or because of missed asthma diagnoses due to reduced health care seeking behaviors during pandemic years. Fifth, because of limited sequencing data, we were not able to account for EV-D68 lineage in our analysis, which may impact illness presentation. Sixth, our data may not be generalizable to other geographic sites because NVSN enrolls participants at only 7 US medical centers. Seventh, NVSN enrollment occurs fewer than 7 days a week, therefore, some EV-D68–associated illnesses may have been missed. Eighth, NVSN enrollment is voluntary, and characteristics of patients enrolled may differ to those not enrolled.

## Conclusions

This cross-sectional study summarizes NVSN systematic EV-D68 surveillance data from 2017 through 2022, generating valuable insight into the epidemiologic and clinical presentation of EV-D68 in children in the US, and informing awareness and preparedness. Clinical and public health practitioners need to be aware that EV-D68 (while still a concern for children with asthma) can cause severe respiratory illness in otherwise healthy children of all ages, and that hospitalized children with nonasthma underlying conditions may be at higher risk for severe outcomes. Additionally, while EV-D68 often presents as an asthma-like illness, other respiratory presentations are also observed. Additional years of active surveillance are critical to fully understand EV-D68 epidemiology as disruptions from the COVID-19 pandemic dissipate, and health care preparation for severe EV-D68 outcomes is prudent.

## Notes

Supplement 1. **eTable 1.** EV-D68 Testing Algorithms by Site, New Vaccine Surveillance Network, 2017-2022 **eTable 2.** Characteristics of All EV-D68-Positive Children Enrolled in New Vaccine Surveillance Network, 2017-2022, by Surveillance Period **eTable 3.** Determination of EV-D68 Clade Using Viral Sequencing From Convenience Samples **eTable 4.** Characteristics of Hospitalized Children With EV-D68 Without Viral Codetection, by Age Group and Reported History of Asthma/RAD, New Vaccine Surveillance Network, 2017-2022 **eTable 5.** Characteristics of Hospitalized Children With EV-D68 Without Viral Codetection, by Receipt of Supplemental Oxygen and Intensive Care, New Vaccine Surveillance Network, 2017-2022 **eTable 6.** Multivariable Comparison of Supplemental Oxygen and Intensive Care Unit Utilization Among Hospitalized Children Aged 2-17 Years With EV-D68, New Vaccine Surveillance Network, 2017-2022 **eTable 7.** Multivariable Comparison of Supplemental Oxygen and Intensive Care Utilization Among Hospitalized Children With EV-D68 (All Ages), Including Children With Viral Codetections; New Vaccine Surveillance Network, 2017-2022

Supplement 2.
Data Sharing Statement
