# Supplementary material for: Enterovirus D68–Associated Respiratory Illness in Children
Source: JAMA Netw Open. 2025 May 8;8(5):e259131. doi: 10.1001/jamanetworkopen.2025.9131 (PMC12062906; doi:10.1001/jamanetworkopen.2025.9131)
Supplement: Supplement 1. — eTable 1. EV-D68 Testing Algorithms by Site, New Vaccine Surveillance Network, 2017-2022 eTable 2. Characteristics of All EV-D68-Positive Children Enrolled in New Vaccine Surveillance Network, 2017-2022, by Surveillance Period eTable 3. Determination of EV-D68 Clade Using Viral Sequencing From Convenience Samples eTable 4. Characteristics of Hospitalized Children With EV-D68 Without Viral Codetection, by Age Group and Reported History of Asthma/RAD, New Vaccine Surveillance Network, 2017-2022 eTable 5. Characteristics of Hospitalized Children With EV-D68 Without Viral Codetection, by Receipt of Supplemental Oxygen and Intensive Care, New Vaccine Surveillance Network, 2017-2022 eTable 6. Multivariable Comparison of Supplemental Oxygen and Intensive Care Unit Utilization Among Hospitalized Children Aged 2-17 Years With EV-D68, New Vaccine Surveillance Network, 2017-2022 eTable 7. Multivariable Comparison of Supplemental Oxygen and Intensive Care Utilization Among Hospitalized Children With EV-D68 (All Ages), Including Children With Viral Codetections; New Vaccine Surveillance Network, 2017-2022 [file jamanetwopen-e259131-s001.pdf]

Supplemental Online Content

Clopper BR, Lopez AS, Goldstein LA, et al. Enterovirus D68–associated respiratory illness in children. *JAMA Netw. Open*. 2025;8(5):e259131. doi:10.1001/jamanetworkopen.2025.9131

eTable 1. EV-D68 Testing Algorithms by Site, New Vaccine Surveillance Network, 2017-2022

eTable 2. Characteristics of All EV-D68-Positive Children Enrolled in New Vaccine Surveillance Network, 2017-2022, by Surveillance Period

eTable 3. Determination of EV-D68 Clade Using Viral Sequencing From Convenience Samples

eTable 4. Characteristics of Hospitalized Children With EV-D68 Without Viral Codetection, by Age Group and Reported History of Asthma/RAD, New Vaccine Surveillance Network, 2017-2022

eTable 5. Characteristics of Hospitalized Children With EV-D68 Without Viral Codetection, by Receipt of Supplemental Oxygen and Intensive Care, New Vaccine Surveillance Network, 2017-2022

eTable 6. Multivariable Comparison of Supplemental Oxygen and Intensive Care Unit Utilization Among Hospitalized Children Aged 2-17 Years With EV-D68, New Vaccine Surveillance Network, 2017-2022

eTable 7. Multivariable Comparison of Supplemental Oxygen and Intensive Care Utilization Among Hospitalized Children With EV-D68 (All Ages), Including Children With Viral Codetections; New Vaccine Surveillance Network, 2017-2022

This supplemental material has been provided by the authors to give readers additional information about their work.

© 2025 Clopper BR et al. *JAMA Network Open*.

eTable 1. EV-D68 Testing Algorithms by Site, New Vaccine Surveillance Network, 2017-2022

Testing Approach	Cincinnati	Houston	Kansas City	Nashville	Pittsburgh	Rochester	Seattle
All ARI specimens are tested with an EV-D68-specific assay.		X[a]		X	X		
All specimens first tested for rhinovirus and/or enterovirus (RV/EV). RV/EV-positive specimens are then tested with an EV-D68-specific assay.	X	X[a]	X			X	X
Abbreviations: ARI = acute respiratory illness							

[a] Beginning May 1, 2022, all Houston specimens were tested using an EV-D68-specific assay, regardless of RV/EV-positivity.

© 2025 Clopper BR et al. *JAMA Network Open*.

eTable 2. Characteristics of All EV-D68-Positive Children Enrolled in New Vaccine Surveillance Network, 2017-2022, by Surveillance Period

	**2017**	**2018**	**2019**	**2020**	**2021**	**2022**	**All years**
	July-Oct	July-Nov	July-Nov	July-Nov	July-Dec	Jan-Dec	
	n (col %)	*n* (col %)	*n* (col %)	*n* (col %)	*n* (col %)	*n* (col %)	*n* (col %)
**Total**	2 (100)	382 (100)	6 (100)	30 (100)	23 (100)	533 (100)	976 (100)
**Age Group (months)**							
Median (IQR)	8.5 (2-15)	2.0 (1-5)	6.5 (0-12)	5.0 (1-9)	3.0 (2-3)	2.0 (1-5)	2.0 (1-5)
0 - 5	0 (0)	24 (6.3)	1 (16.7)	4 (13.3)	0 (0)	24 (4.5)	53 (5.4)
6 - 11	0 (0)	30 (7.9)	1 (16.7)	3 (10.0)	2 (8.7)	52 (9.8)	88 (9.0)
12 - 23	0 (0)	90 (23.6)	0 (0)	2 (6.7)	1 (4.4)	119 (22.3)	212 (21.7)
24 - 59	1 (50)	139 (36.4)	1 (16.7)	5 (16.7)	16 (69.6)	197 (37.0)	359 (36.8)
5-11 y	0 (0)	85 (22.3)	1 (16.7)	10 (33.3)	4 (17.4)	119 (22.3)	219 (22.4)
12-17 y	1 (50)	14 (3.7)	2 (33.3)	6 (20.0)	0 (0)	22 (4.1)	45 (4.6)
**NVSN Site**							
Nashville	0 (0)	47 (12.3)	1 (16.7)	6 (20.0)	3 (13.0)	77 (14.5)	134 (13.7)
Rochester, NY	1 (50)	63 (16.5)	0 (0)	1 (3.3)	1 (4.4)	34 (6.4)	100 (10.3)
Cincinnati	0 (0)	56 (14.7)	0 (0)	3 (10.0)	0 (0)	67 (12.6)	126 (12.9)
Seattle	0 (0)	38 (10)	2 (33.3)	0 (0)	0 (0)	76 (14.3)	116 (11.9)
Houston	1 (50)	28 (7.3)	2 (33.3)	3 (10.0)	14 (60.9)	75 (14.1)	123 (12.6)
Kansas City	0 (0)	54 (14.1)	1 (16.7)	16 (53.3)	0 (0)	81 (15.2)	152 (15.6)
Pittsburgh	0 (0)	96 (25.1)	0 (0)	1 (3.3)	5 (21.7)	123 (23.1)	225 (23.1)
**Month**							
July	0 (0)	16 (4.2)	1 (16.7)	1 (3.3)	0 (0)	47 (8.8)	65 (6.7)
August	0 (0)	77 (20.2)	0 (0)	3 (10.0)	2 (8.7)	176 (33.0)	258 (26.4)
September	0 (0)	169 (44.2)	2 (33.3)	11 (36.7)	1 (4.4)	195 (36.6)	378 (38.7)
October	2 (100)	96 (25.1)	1 (16.7)	13 (43.3)	3 (13.0)	30 (5.6)	145 (14.9)
November	0 (0)	24 (6.3)	2 (33.3)	2 (6.7)	6 (26.1)	3 (5.6)	37 (3.8)
December	NA	NA	NA	NA	11 (47.8)	0 (0)	11 (1.1)
January-June	NA	NA	NA	NA	NA	82 (15.4)	82 (8.4)
**Any Viral Co-Detection[a]**	0 (0)	34 (8.9)	1 (16.7)	0 (0)	3 (13.0)	82 (15.4)	120 (12.3)
**Any underlying condition (including asthma/RAD history)**	2 (100)	177 (46.3)	2 (33.3)	17 (56.7)	9 (39.1)	185 (34.7)	392 (40.2)
**Any underlying condition (excluding asthma/RAD history)**	1 (50)	44 (11.5)	0 (0)	2 (6.7)	5 (21.7)	61 (11.4)	113 (11.6)

© 2025 Clopper BR et al. *JAMA Network Open*.

**History of asthma/RAD**	1 (50)	139 (36.4)	2 (33.3)	14 (46.7)	5 (21.7)	127 (23.8)	288 (29.5)
**Final Level of Care**							
ED	1 (50)	125 (32.7)	1 (16.7)	15 (50.0)	8 (34.8)	214 (40.2)	364 (37.3)
Inpatient	1 (50)	257 (67.3)	5 (83.3)	15 (50.0)	15 (65.2)	319 (59.9)	612 (62.7)
Abbreviations: RAD = Reactive Airway Disease; y = years							

Note: Percentages may not add to 100 due to rounding.
[a] Co-detected viruses included: RSV, influenza, HMPV, HPIV1–4, adenovirus, seasonal HCoVs, SARS-CoV-2

© 2025 Clopper BR et al. *JAMA Network Open*.

eTable 3. Determination of EV-D68 Clade Using Viral Sequencing From Convenience Samples

Year	EV-D68 Sequencing attempts	Clade	A2/D	B1	B2	B3
2018	60		0	0	0	42
2019	0		-	-	-	-
2020	25		15	0	0	0
2021	15		0	0	0	5
2022	223		0	0	0	159

© 2025 Clopper BR et al. *JAMA Network Open*.

eTable 4. Characteristics of Hospitalized Children With EV-D68 Without Viral Codetection, by Age Group and Reported History of Asthma/RAD, New Vaccine Surveillance Network, 2017-2022

	All ages		<2 years		2-4 years		5-17 years	
**Asthma History Status**	**No Asthma**	**Asthma**	**No Asthma**	**Asthma**	**No Asthma**	**Asthma**	**No Asthma**	**Asthma**
	n (%)	*n* (%)	*n* (%)	*n* (%)	*n* (%)	*n* (%)	*n* (%)	*n* (%)
**Total**	337 (100)	199 (100)	156 (100)	17 (100)	114 (100)	81 (100)	67 (100)	101 (100)
**Age median (IQR)**	2.0 (1-4) y	5.0 (3-7) y	14 (9-19) m	16 (15-22) m	39 (29-48) m	42 (35-49) m	6.0 (5-8) y	7.0 (6-10) y
**Non-asthma underlying medical conditions**								
Any underlying condition (excluding asthma/RAD)	59 (17.5)	18 (9.1)	24 (15.4)	2 (11.8)	20 (17.5)	9 (11.1)	15 (22.4)	7 (6.9)
Chronic Lung Disease (excluding asthma/RAD)[a]	18 (5.3)	0 (0)	6 (3.9)	0 (0)	8 (7.0)	0 (0)	4 (6.0)	0 (0)
Neuromuscular Disease[b]	25 (7.4)	6 (3.0)	6 (3.9)	2 (11.8)	9 (7.9)	4 (4.9)	10 (14.9)	0 (0)
Immunocompromise[c]	3 (0.9)	3 (1.5)	2 (1.3)	0 (0)	1 (0.9)	0 (0)	0 (0)	3 (3.0)
Heart Condition[d]	15 (4.5)	4 (2.0)	7 (4.5)	0 (0)	6 (5.3)	3 (3.7)	2 (3.0)	1 (1.0)
Developmental Disorder[e]	22 (6.5)	8 (4.0)	3 (1.9)	2 (11.8)	10 (8.8)	4 (4.9)	9 (13.4)	2 (2.0)
**Symptoms reported[f]**								
Cough	325 (96.4)	193 (97.0)	146 (93.6)	17 (100)	114 (100)	80 (98.8)	65 (97.0)	96 (95.1)
Nasal congestion	294 (87.2)	147 (73.9)	143 (91.7)	15 (88.2)	99 (86.8)	61 (75.3)	52 (77.6)	71 (70.3)
Wheezing	239 (70.9)	179 (90.0)	96 (61.5)	14 (82.4)	93 (81.6)	75 (92.6)	50 (74.6)	90 (89.1)
Fever	202 (59.9)	93 (46.7)	92 (59.0)	8 (47.1)	72 (63.2)	42 (51.9)	38 (56.7)	43 (42.6)
Ear pain	36 (10.7)	14 (7.0)	20 (12.8)	1 (5.9)	11 (9.7)	4 (4.9)	5 (7.5)	9 (8.9)
Sore throat	78 (23.2)	62 (31.2)	23 (14.7)	2 (11.8)	27 (23.7)	13 (16.1)	28 (41.8)	47 (46.5)
Vomiting	62 (18.4)	27 (13.6)	30 (19.2)	1 (5.9)	17 (14.9)	14 (17.3)	15 (22.4)	12 (11.9)
Muscle pain	24 (7.1)	30 (15.1)	4 (2.6)	2 (11.8)	11 (9.7)	12 (14.8)	9 (13.4)	16 (15.8)
Rapid or shallow breathing/shortness of breath	309 (91.7)	194 (97.5)	139 (89.1)	17 (100)	106 (93.0)	78 (96.3)	64 (95.5)	99 (98.0)
**Severity of Illness**								
LOS median (IQR)	1 (1-2)	1 (1-2)	1 (1-2)	1 (1-2)	1 (1-2)	1 (1-1)	1 (1-2)	1 (1-2)
Respiratory Support (supplemental oxygen)	218 (64.7)	121 (60.8)	101 (64.7)	12 (70.6)	73 (64.0)	46 (56.8)	44 (65.7)	63 (62.4)
ICU	60 (17.8)	27 (13.6)	28 (18.0)	4 (23.5)	18 (15.8)	9 (11.1)	14 (20.9)	14 (13.9)
Respiratory Support (intubation)	11 (3.3)	0 (0)	5 (3.2)	0 (0)	4 (3.5)	0 (0)	2 (3.0)	0 (0)
**Asthma-related discharge diagnosis[g]**								
Primary asthma/RAD	106 (31.5)	160 (80.4)	15 (9.6)	7 (41.2)	56 (49.1)	68 (84.0)	35 (52.2)	85 (84.2)
Any asthma/RAD	148 (43.9)	185 (93.0)	30 (19.2)	12 (70.6)	71 (62.3)	74 (91.4)	47 (70.2)	99 (98.0)

© 2025 Clopper BR et al. *JAMA Network Open*.

Alternate specific	118 (35.0)	174 (87.4)	17 (10.9)	11 (64.7)	58 (50.9)	68 (84.0)	43 (64.2)	95 (94.1)
**Non-asthma discharge diagnoses**								
Upper respiratory infection[h]	23 (6.8)	1 (0.5)	8 (5.1)	1 (5.9)	10 (8.8)	0 (0)	5 (7.5)	0 (0)
Bronchiolitis (J21)	71 (21.1)	2 (1.0)	66 (42.3)	2 (11.8)	5 (4.4)	0 (0)	0 (0)	0 (0)
Respiratory signs and symptoms[i]	31 (9.2)	7 (3.5)	14 (9.0)	4 (23.5)	13 (11.4)	1 (1.2)	4 (6.0)	2 (2.0)
Wheezing only (R06.2)	13 (3.9)	1 (0.5)	6 (3.9)	0 (0)	6 (5.3)	0 (0)	1 (1.5)	1 (1.0)
Pneumonia, non-TB[j]	22 (6.5)	15 (7.5)	4 (2.6)	0 (0)	11 (9.7)	6 (7.4)	7 (10.5)	9 (8.9)
Abbreviations: IQR = interquartile range; LOS = length of stay; m = months; RAD = Reactive Airway Disease; TB = tuberculosis; y = years								

Note: Cases with viral co-detections were excluded. Percentages may not add to 100 due to rounding.

[a] Cystic fibrosis, bronchopulmonary dysplasia, chronic lung disease of prematurity, or other chronic lung condition.

[b] Cerebral palsy, seizure disorder, or other neurologic or neuromuscular disorder.

[c] Immune condition, transplant recipient (peripheral blood stem cells, bone marrow, cord blood, or organ), cancer, and sickle cell anemia.

[d] Congenital heart malformation or other heart condition.

[e] Developmental disorders including intellectual disability, Pervasive Developmental Disorder, Global Developmental Delay, Autism Spectrum Disorder, or other developmental disorder.

[f] These data were collected during the parent/guardian interview. The researchers' ability to capture certain symptoms may be dependent on age of the child.

[g] Primary asthma-related discharge diagnosis included ICD-10-CM code J45 in the primary position. Any asthma-related discharge diagnosis included ICD-10-CM code J45 in any primary or secondary discharge diagnosis code position (up to 10). The alternate specific definition included cases with discharge diagnosis code 'J45' and the following sub-specifications in any diagnosis code position (primary or secondary): .01, .02, .11, .12, .21, .22, .31, .32, .41, .42, .51, .52, .901, .902.

[h] ICD-10-CM codes included: J02.0 (streptococcal pharyngitis), J02.8 (acute pharyngitis due to other unspecified organisms), J02.9 (acute pharyngitis, unspecified), J04.10 (acute tracheitis without obstruction), J05.0 (acute obstructive laryngitis), J06.9 (acute upper respiratory infection, unspecified).

[i] ICD-10-CM codes included: R05 (cough), R05.8 (other specified cough), R05.9 (cough, unspecified), R06.00 (unspecified dyspnea), R06.02 (shortness of breath), R06.2 (wheezing), R06.03 (acute respiratory distress), R06.89 (other abnormalities of breathing), R06.9 (unspecified abnormalities of breathing), R09.02 (hypoxemia), and R09.81 (nasal congestion).

[j] ICD-10-CM codes included: J12.3 (human metapneumovirus pneumonia), J12.89 (other viral pneumonia), J12.9 (viral pneumonia, unspecified), J15.7 (pneumonia due to mycoplasma pneumoniae), J15.9 (unspecified bacterial pneumonia), J16.8 (pneumonia due to other specified infectious organisms), J18.1 (lobar pneumonia, unspecified organism), J18.8 (other pneumonia, organism unspecified), J18.9 (pneumonia, unspecified organism).

© 2025 Clopper BR et al. *JAMA Network Open*.

eTable 5. Characteristics of Hospitalized Children With EV-D68 Without Viral Codetection, by Receipt of Supplemental Oxygen and Intensive Care, New Vaccine Surveillance Network, 2017-2022

	All	No supplemental oxygen	Supplemental oxygen received	p value[a]	No ICU	ICU	p value[a]
**Total** (row%)	536 (100)	197 (36.8)	339 (63.2)		449 (83.8)	87 (16.2)	
**Age Group**	*n* (col%)	*n* (col%)	*n* (col%)		*n* (col%)	*n* (col%)	
Median in months (IQR)	40 (19-69)	42 (20-67)	40 (19-69)	0.96[b]	40 (20–69)	38 (17–66)	0.75[b]
0 - 5 months	19 (3.5)	13 (6.6)	6 (1.8)	**0.04**	17 (3.8)	2 (2.3)	0.69
6 - 11 months	39 (7.3)	10 (5.1)	29 (8.6)		32 (7.1)	7 (8.1)	
12 – 23 months	115 (21.5)	37 (18.8)	78 (23.0)		92 (20.5)	23 (26.4)	
24 – 59 months	195 (36.4)	76 (38.6)	119 (35.1)		168 (37.4)	27 (31)	
5-11 years	145 (27.1)	53 (26.9)	92 (27.1)		122 (27.2)	23 (26.4)	
12-17 years	23 (4.3)	8 (4.1)	15 (4.4)		18 (4.0)	5 (5.8)	
**Sex**							
Female	206 (38.4)	72 (36.6)	134 (39.5)	0.49	163 (36.3)	43 (49.4)	**0.02**
Male	330 (61.6)	125 (63.5)	205 (60.5)		286 (63.7)	44 (50.6)	
**Race and Ethnicity**							
Black, non-Hispanic	182 (34)	82 (41.6)	100 (29.5)	**0.04**	153 (34.1)	29 (33.3)	0.99
Hispanic	94 (17.5)	27 (13.7)	67 (19.8)		78 (17.4)	16 (18.4)	
White, non-Hispanic	199 (37.1)	70 (35.5)	129 (38.1)		168 (37.4)	31 (35.6)	
Other, non-Hispanic	55 (10.3)	17 (8.6)	38 (11.2)		45 (10.0)	10 (11.5)	
Unknown	6 (1.1)	1 (0.5)	5 (1.5)		5 (1.1)	1 (1.2)	
**Surveillance year**							
2017 (Jul-Oct)	1 (0.2)	0 (0)	1 (0.3)	**<0.001**[c]	0 (0)	1 (1.2)	0.07[c]
2018 (Jul-Nov)	233 (43.5)	103 (52.3)	130 (38.4)		200 (44.5)	33 (37.9)	
2019 (Jul-Nov)	4 (0.8)	1 (0.5)	3 (0.9)		2 (0.5)	2 (2.3)	
2020 (Jul-Nov)	15 (2.8)	5 (2.5)	10 (3.0)		12 (2.7)	3 (3.5)	
2021 (Jul-Dec)	13 (2.4)	0 (0)	13 (3.8)		12 (2.7)	1 (1.2)	
2022 (Jan-Dec)	270 (50.4)	88 (44.7)	182 (53.7)		223 (49.7)	47 (54.0)	
**Surveillance period**[d]							
Pre-pandemic	238 (44.4)	104 (52.8)	134 (39.5)	**0.003**	202 (45.0)	36 (41.4)	0.54
During pandemic	298 (55.6)	93 (47.2)	205 (60.5)		247 (55.0)	51 (58.6)	
**NVSN Site**							

© 2025 Clopper BR et al. *JAMA Network Open*.

Nashville	65 (12.1)	29 (14.7)	36 (10.6)		53 (11.8)	12 (13.8)	
Rochester, NY	64 (11.9)	30 (15.2)	34 (10.0)		57 (12.7)	7 (8.1)	
Cincinnati	67 (12.5)	39 (19.8)	28 (8.3)		58 (12.9)	9 (10.3)	
Seattle	55 (10.3)	24 (12.2)	31 (9.1)	**<.001**	46 (10.2)	9 (10.3)	0.13
Houston	77 (14.4)	10 (5.1)	67 (19.8)		56 (12.5)	21 (24.1)	
Kansas City	67 (12.5)	15 (7.6)	52 (15.3)		59 (13.1)	8 (9.2)	
Pittsburgh	141 (26.3)	50 (25.4)	91 (26.8)		120 (26.7)	21 (24.1)	
**Underlying Medical Conditions[e]**							
No underlying condition	268 (50.0)	106 (53.8)	162 (47.8)	0.18	234 (52.1)	34 (39.1)	**0.03**
≥1 underlying condition	268 (50.0)	91 (46.2)	177 (52.2)		215 (47.9)	53 (60.9)	
Any underlying condition (excluding asthma/RAD)	77 (14.4)	14 (7.1)	63 (18.6)	**<0.001**	51 (11.4)	26 (29.9)	**<0.001**
Asthma/RAD	199 (37.1)	78 (39.6)	121 (35.7)	0.37	172 (38.3)	27 (31.0)	0.20
Asthma/RAD (among 2-17 year-olds only)	182 (50.1)	73 (53.3)	109 (48.2)	0.35	159 (51.6)	23 (41.8)	0.18
Chronic Lung Disease (excluding asthma/RAD)[f]	18 (3.4)	1 (0.5)	17 (5.0)	**0.005**	5 (1.1)	13 (14.9)	**<0.001**
Neuromuscular Disease[g]	31 (5.8)	4 (2.0)	27 (8.0)	**0.005**	19 (4.2)	12 (13.8)	**0.001**
Immunocompromise[h]	6 (1.1)	1 (0.5)	5 (1.5)	0.42[c]	5 (1.1)	1 (1.2)	1.00[c]
Heart Condition[i]	19 (3.5)	1 (0.5)	18 (5.3)	**0.004**	14 (3.1)	5 (5.8)	0.23
Developmental Disorder[j]	30 (5.6)	5 (4.4)	25 (11.8)	**0.03**	21 (7.8)	9 (15.5)	0.07

Abbreviations: ICU = Intensive Care Unit; RAD = Reactive Airway Disease
Study participants indicated “Other, non-Hispanic” race and ethnicity voluntarily if they did not self-identify as Black, non-Hispanic; Hispanic; or White, non-Hispanic race and ethnicity.

Note: Cases with viral co-detections were excluded. Percentages may not add to 100 due to rounding.
[a] Pearson’s chi-squared comparisons were used to compare demographic and clinical characteristics by receipt of supplemental oxygen and receipt of ICU care.
[b] Results using Wilcoxon Rank Sum test where data were not normally distributed.
[c] Results using Fisher's Exact test.
[d] Pre-pandemic includes surveillance periods from July 2017–February 2020. Pandemic includes surveillance periods from March 2020–December 2022.
[e] Underlying conditions included: congenital heart malformation or other heart condition, transplant recipient, cancer, sickle cell anemia, cerebral palsy, seizure disorder or other neurologic or neuromuscular disorder, asthma/reactive airway disease, cystic fibrosis, bronchopulmonary dysplasia, chronic lung disease of prematurity or other chronic lung condition, kidney disease, Down syndrome or other genetic or metabolic disorder, blood disorders, liver disease, diabetes, chronic endocrine condition, chronic gastrointestinal disease, and other developmental disabilities.
[f] Cystic fibrosis, bronchopulmonary dysplasia, chronic lung disease of prematurity, or other chronic lung condition.
[g] Cerebral palsy, seizure disorder, or other neurologic or neuromuscular disorder.
[h] Immune condition, transplant recipient (peripheral blood stem cells, bone marrow, cord blood, or organ), cancer, and sickle cell anemia.
[i] Congenital heart malformation or other heart condition.
[j] Developmental disorders including intellectual disability, Pervasive Developmental Disorder, Global Developmental Delay, Autism Spectrum Disorder, or other developmental disorder.

© 2025 Clopper BR et al. *JAMA Network Open*.

eTable 6. Multivariable Comparison of Supplemental Oxygen and Intensive Care Unit Utilization Among Hospitalized Children Aged 2-17 Years With EV-D68, New Vaccine Surveillance Network, 2017-2022[a]

	Supplemental Oxygen			OR (95% CI)	aOR (95% CI)	Intensive Care Unit			OR (95%CI)	aOR (95% CI)
	no	*yes*	row %			*no*	*yes*	row %		
**Total**	137	226	62.3			308	55	15.2		
**Surveillance Period[b]**										
Pre-pandemic	73	85	53.8	REF	REF	135	23	14.6	REF	REF
Pandemic	64	141	68.8	**1.89 (1.23-2.91)**	**1.85 (1.14-3.00)**	173	32	15.6	1.09 (0.61-1.94)	1.00 (0.52-1.92)
**Age Group (months)**										
24 < 60	76	119	61	0.89 (0.58-1.37)	0.91 (0.56-1.46)	168	27	13.9	0.80 (0.45-1.43)	0.71 (0.38-1.33)
≥60	61	107	63.7	REF	REF	140	28	16.7	REF	REF
**Sex**										
Female	55	97	63.8	1.12 (0.73-1.73)	1.14 (0.71-1.84)	122	30	19.7	**1.83 (1.03-3.26)**	**2.00 (1.08-3.72)**
Male	82	129	61.1	REF	REF	186	25	11.9	REF	REF
**Race/Ethnicity**										
Black, non-Hispanic	64	74	53.6	**0.57 (0.35-0.94)**	**0.55 (0.31-0.98)**	117	21	15.2	1.06 (0.53-2.09)	1.15 (0.53-2.48)
Hispanic	19	43	69.4	1.12 (0.58-2.16	0.60 (0.26-1.37)	53	9	14.5	1.00 (0.42-2.38)	0.55 (0.18-1.72)
White, non-Hispanic	41	83	66.9	REF	REF	106	18	14.5	REF	REF
Other, non-Hispanic	12	24	66.7	0.99 (0.45-2.17)	1.25 (0.53-2.94)	29	7	19.4	1.42 (0.54-3.73)	1.54 (0.54-4.41)
Unknown	1	2	66.7	0.99 (0.09-11.2)	1.14 (0.10-13.54)	3	0	0	NA	NA
**NVSN Site**										
Nashville	21	22	51.2	**0.47 (0.23-0.97)**	**0.45 (0.20-0.97)**	37	6	14	0.78 (0.29-2.11)	0.75 (0.26-2.16)
Rochester, NY	22	26	54.2	0.53 (0.26-1.06)	0.71 (0.34-1.50)	42	6	12.5	0.68 (0.25-1.85)	0.87 (0.30-2.49)
Cincinnati	28	14	33.3	**0.22 (0.10-0.48)**	**0.32 (0.14-0.71)**	37	5	11.9	0.65 (0.22-1.87)	0.68 (0.22-2.14)
Seattle	15	18	54.6	0.53 (0.24-1.19)	**0.42 (0.18-0.99)**	31	2	6.1	0.31 (0.07-1.41)	0.28 (0.06-1.37)
Houston	7	42	85.7	**2.67 (1.08-6.57)**	2.82 (0.98-8.14)	36	13	26.5	1.73 (0.77-3.89)	1.85 (0.62-5.54)
Kansas City	12	32	72.7	1.19 (0.54-2.59)	1.78 (0.76-4.18)	39	5	11.4	0.61 (0.21-1.77)	0.61 (0.19-1.94)
Pittsburgh	32	72	69.2	REF	REF	86	18	17.3	REF	REF
**Any underlying medical conditions (excluding asthma/RAD)**										
No	128	184	59	REF	REF	274	38	12.2	REF	REF
Yes	9	42	82.4	**3.25 (1.53-6.90)**	**2.92 (1.31-6.55)**	34	17	33.3	**3.61 (1.84-7.07)**	**3.46 (1.65-7.27)**
**History of asthma/RAD**										

© 2025 Clopper BR et al. *JAMA Network Open*.

No	64	117	64.6	REF	REF	149	32	17.7	REF	REF
Yes	73	109	59.9	0.82 (0.53-1.25)	1.22 (0.74-2.01)	159	23	12.6	0.67 (0.38-1.20)	0.84 (0.44-1.62)

Abbreviations: aOR = adjusted odds ratio; NVSN = New Vaccine Surveillance Network; OR = odds ratio (unadjusted); RAD = Reactive Airway Disease
Study participants indicated “Other, non-Hispanic” race and ethnicity voluntarily if they did not self-identify as Black, non-Hispanic; Hispanic; or White, non-Hispanic race and ethnicity.

Note: Cases with viral co-detections were excluded.

[a] Each outcome adjusted for surveillance period, age group, sex, race/ethnicity, NVSN site, presence of any underlying medical condition (excluding asthma/RAD), and history of asthma/RAD.

[b] Pre-pandemic period is defined as: 2017 – Feb. 29, 2020; Pandemic period is defined as March 1, 2020–2022.

© 2025 Clopper BR et al. *JAMA Network Open*.

eTable 7. Multivariable Comparison of Supplemental Oxygen and Intensive Care Utilization Among Hospitalized Children With EV-D68 (All Ages), Including Children With Viral Codetections; New Vaccine Surveillance Network, 2017-2022[a]

	Supplemental Oxygen			OR (95% CI)	aOR (95% CI)	Intensive Care Unit			OR (95% CI)	aOR (95% CI)
	no	*yes*	row %			*no*	*yes*	row %		
**Total**	227	385	62.9			507	105	17.2		
**Surveillance Period[b]**										
Pre-pandemic	115	148	56.3	REF	REF	221	42	16	REF	REF
Pandemic	112	237	67.9	**1.64 (1.18-2.29)**	**1.49 (1.03-2.14)**	286	63	18.1	1.16 (0.76-1.78)	0.95 (0.60-1.53)
**Age Group (months)**										
0 < 12	28	44	61.1	0.89 (0.51-1.56)	0.75 (0.39-1.47)	57	15	20.8	1.32 (0.66-2.63)	1.00 (0.46-2.19)
12 < 24	51	93	64.6	1.03 (0.65-1.63)	1.10 (0.64-1.89)	116	28	19.4	1.21 (0.68-2.13)	1.14 (0.60-2.17)
24 < 60	83	133	61.6	0.91 (0.60-1.36)	0.94 (0.60-1.46)	184	32	14.8	0.87 (0.51-1.50)	0.85 (0.48-1.51)
≥60	65	115	63.9	REF	REF	150	30	16.7	REF	REF
**Sex**										
Female	83	157	65.4	1.20 (0.85-1.68)	1.10 (0.77-1.59)	188	52	21.7	**1.67 (1.09-2.54)**	**1.60 (1.02-2.48)**
Male	144	228	61.3	REF	REF	319	53	14.3	REF	REF
**Race/Ethnicity**										
Black, non-Hispanic	93	110	54.2	**0.66 (0.45-0.97)**	**0.62 (0.40-0.96)**	170	33	16.3	1.02 (0.61-1.71)	1.19 (0.67-2.10)
Hispanic	30	80	72.7	1.49 (0.90-2.44)	0.82 (0.45-1.49)	87	23	20.9	1.39 (0.78-2.49)	0.94 (0.46-1.91)
White, non-Hispanic	83	149	64.2	REF	REF	195	37	16	REF	REF
Other, non-Hispanic	19	41	68.3	1.20 (0.66-2.21)	1.33 (0.71-2.51)	49	11	18.3	1.18 (0.56-2.49)	1.23 (0.57-2.68)
Unknown	2	5	71.4	1.39 (0.26-7.34)	1.38 (0.25-7.61)	6	1	14.3	0.88 (0.10-7.51)	0.71 (0.07-6.90)
**NVSN Site**										
Nashville	32	43	57.3	0.82 (0.47-1.42)	0.81 (0.45-1.46)	59	16	21.3	1.58 (0.78-3.19)	1.60 (0.77-3.34)
Rochester, NY	32	36	52.9	0.68 (0.39-1.21)	0.85 (0.46-1.55)	61	7	10.3	0.67 (0.27-1.64)	0.75 (0.30-1.91)
Cincinnati	45	35	43.8	**0.47 (0.28-0.81)**	0.60 (0.34-1.06)	67	13	16.3	1.13 (0.54-2.36)	1.11 (0.51-2.40)
Seattle	28	36	56.3	0.78 (0.44-1.40)	0.64 (0.34-1.18)	54	10	15.6	1.08 (0.49-2.41)	1.03 (0.44-2.40)
Houston	11	77	87.5	**4.26 (2.10-8.62)**	**4.07 (1.84-8.99)**	61	27	30.7	**2.58 (1.38-4.83)**	**2.32 (1.08-5.00)**
Kansas City	17	56	76.7	**2.00 (1.07-3.75)**	**2.35 (1.22-4.55)**	65	8	11	0.72 (0.31-1.68)	0.68 (0.28-1.65)
Pittsburgh	62	102	62.2	REF	REF	140	24	14.6	REF	REF
**Any viral codetection**										
No	197	339	63.3	REF	REF	449	87	16.2	REF	REF
Yes	30	46	60.5	0.89 (0.55-1.46)	0.87 (0.51-1.49)	58	18	23.7	1.60 (0.90-2.85)	1.52 (0.82-2.80)

© 2025 Clopper BR et al. *JAMA Network Open*.

**Any underlying medical conditions (excluding asthma/RAD)**										
No	208	316	60.3	REF	REF	447	77	14.7	REF	REF
Yes	19	69	78.4	**2.39 (1.40-4.09)**	**2.06 (1.17-3.65)**	60	28	31.8	**2.71 (1.63-4.51)**	**2.31 (1.34-3.98)**
**History of asthma/RAD**										
No	138	251	64.5	REF	REF	315	74	19	REF	REF
Yes	89	134	60.1	0.83 (0.59-1.16)	1.03 (0.68-1.56)	192	31	13.9	0.69 (0.44-1.09)	0.77 (0.45-1.31)
Abbreviations: NVSN = New Vaccine Surveillance Network; RAD = Reactive Airway Disease<br>Study participants indicated "Other, non-Hispanic" race and ethnicity voluntarily if they did not self-identify as Black, non-Hispanic; Hispanic; or White, non-Hispanic race and ethnicity.										

Note: Percentages may not add to 100 due to rounding.

[a] Each outcome adjusted for surveillance period, age group, sex, race/ethnicity, NVSN site, presence of any viral codetection, presence of any underlying medical condition (excluding asthma/RAD), and history of asthma/RAD.

[b] Pre-pandemic period is defined as: 2017 – Feb. 29, 2020; Pandemic period is defined as March 1, 2020–2022.

© 2025 Clopper BR et al. *JAMA Network Open*.
