# Supplementary material for: Enterovirus D68–Associated Respiratory Illness in Children
Source: JAMA Netw Open. 2025 May 8;8(5):e259131. doi: 10.1001/jamanetworkopen.2025.9131 (PMC12062906; doi:10.1001/jamanetworkopen.2025.9131)
Supplement: Supplement 2. — Data Sharing Statement [file jamanetwopen-e259131-s002.pdf]

Data Sharing Statement

Clopper. Enterovirus D68–Associated Respiratory Illness in Children. *JAMA Netw Open*. Published May 08, 2025. doi:10.1001/jamanetworkopen.2025.9131

Data
Data available: No

Additional Information
Explanation for why data not available: Data can be made available upon request to corresponding author in accordance with network steering committee guidelines
